# Fecal Microbiota Changes in Angus Beef Cows Persistently Infected by Bovine Viral Diarrhea Virus

**DOI:** 10.3390/vetsci12060538

**Published:** 2025-06-02

**Authors:** Ruiyang Xia, Yalu Chen, Pengfei Yi, Yawei Sun, Lijing Chen, Xuelian Ma, Qi Zhong, Na Li, Gang Yao

**Affiliations:** 1 College of Veterinary Medicine, Xinjiang Agricultural University, Urumqi 830052, China; xry01131852@163.com (R.X.); 320230073@stu.xjau.edu.cn (P.Y.); syw2008@xjau.edu.cn (Y.S.); 320200055@stu.xjau.edu.cn (L.C.); maxuelian@xjau.edu.cn (X.M.); 2 Animal Disease Control and Prevention Center, Bole 833400, China; 17690569607@163.com; 3 Institute of Veterinary Research, Xinjiang Academy of Animal Sciences, Urumqi 830011, China; yyyzqok@yahoo.com.cn

**Keywords:** Angus beef cow, bovine viral diarrhea virus, persistently infected cow, gut microbiota, 16S rRNA sequencing

## Abstract

Bovine viral diarrhea virus (BVDV) remains a major cause of calf diarrhea with substantial economic impacts on global cattle production. While emerging evidence suggests that the host microbiota may modulate viral infection processes, the specific gut microbial alterations induced by BVDV infection require elucidation. This study investigated gut microbiota composition and functional changes in BVDV-infected cows through 16S rRNA sequencing. Following the epidemiological screening of a large Angus cattle herd using RT-PCR, we identified four persistently infected (PI) animals and four BVDV-negative controls. Fecal analysis revealed Firmicutes and Bacteroidetes as dominant phyla in both groups. A significant increase in the *Ruminococcus* genus was observed in PI cows (*p* < 0.05), with LEfSe analysis identifying *Paludibacter* as a BVDV-associated biomarker. The PI group exhibited reduced alpha diversity (Faith’s PD index, *p* < 0.05) and elevated histidine metabolism pathway abundance compared to controls (*p* < 0.05). These findings demonstrate that persistent BVDV infection induces structural and functional modifications in the bovine gut microbiota, particularly through the enrichment of specific bacterial taxa and altered metabolic potential. The results provide new insights into virus–microbiota interactions, establishing a foundation for understanding how BVDV infection may influence host physiology through microbial community changes.

## Simple Summary

Bovine viral diarrhea virus (BVDV) severely impacts cattle health and farm economies through persistent infections. This study compared the gut microbiota of cows persistently infected (PI) with BVDV to uninfected controls and rigorously confirmed the results via ELISA and PCR to exclude both transient and chronic infections. Persistent BVDV infection altered the gut microbiota, reducing microbial diversity and driving inflammation-associated dysbiosis. PI cows showed reduced microbial diversity, with fewer beneficial bacteria, including *Ruminococcus*, and more inflammation-linked *Paludibacter*. Increased histidine metabolism activity—a pathway tied to inflammation—was observed in PI cows. These changes suggest that BVDV weakens gut health, potentially lowering disease resistance. The findings highlight the importance of balancing the gut microbiota in cattle health, providing actionable insights for managing BVDV. By understanding how viruses alter gut ecosystems, new strategies to improve herd resilience against BVDV infections could be developed.

## 1. Introduction

Bovine viral diarrhea virus (BVDV), a globally prevalent pathogen in cattle populations, causes significant digestive system disorders, including viral diarrhea–mucosal disease [1]. BVDV infection can manifest as inflammatory diarrhea, bloody stools, or anorexia in clinical cases, with mortality occurring in severe infections [2]. This single-stranded RNA virus belongs to the genus *Pestivirus* within the family *Flaviviridae* and exists in two biotypes: cytopathogenic (CP) and non-cytopathogenic (NCP) [3]. Of particular epidemiological importance, NCP-BVDV infection during early gestation (<125 days) results in persistently infected (PI) offspring that continuously shed the virus through bodily secretions, perpetuating herd transmission if undetected [4,5]. Both persistent infection and immune modulation by BVDV contribute to substantial economic losses through reduced productivity, morbidity, and mortality [6].

The gut microbiota plays crucial roles in maintaining host physiology, immunity, and disease resistance across species [7]. For example, *Lactobacillus yeonis* alleviates bacterial diarrhea in yak calves while increasing beneficial bacterial genera in their gut microbiota [8]. Viral infections frequently induce microbial dysbiosis, representing critical aspects of virus–host interactions [9]. A study by Uchiyama et al. demonstrated that bovine leukemia virus (BLV) infection alters the gut microbiota in dairy cattle, enriching rumen fermentation-related taxa such as *Lachnospiraceae* and *Veillonellaceae* in uninfected individuals, while *Haemophilus* abundance negatively correlates with BLV transmission capacity [10]. Similarly, Zika virus (a *Flaviviridae* family member like BVDV) infection reduces Actinobacteria and Firmicutes populations while elevating *Deinococcaceae* and *Spirochaetaceae* levels [11]. In bovine diarrhea research, dysbiosis in diarrheic calves is characterized by elevated *Enterobacteriaceae* and disrupted phage interactions [12], while protective *Lactobacillus* species such as *L. reuteri* show negative correlations with pathogens in healthy calves [13].

Despite established connections between BVDV infection and immune modulation [14], as well as the recognized interplay between gut microbiota and host immunity [15], the microbial changes in PI cattle remain poorly characterized. This knowledge gap persists despite the central role played by PI animals in BVDV epidemiology and the control strategies that require their identification and removal [5]. Here, we conducted epidemiological screening in a large Angus cattle herd to compare gut microbiota composition between BVDV-PI and healthy cows. Our investigation aims to identify PI-associated microbial alterations and provide critical insights for developing targeted BVDV control measures.

## 2. Materials and Methods

### 2.1. Epidemiological Survey and Diagnosis of BVDV-Persistent Infection

#### 2.1.1. Epidemiological Survey

An epidemiological investigation of BVDV was conducted in two large-scale beef cattle farms in northern Xinjiang, China (Farm A: 3856 cows; Farm B: 454 cows). Blood samples were collected from the tail veins of all 4310 cows and sera were separated and stored at −20 °C. Initial antigen screening using the IDEXX BVDV Ag ELISA test kit (IDEXX Laboratories, Westbrook, ME, USA) revealed a positivity rate of 0.44%. Retesting 3 weeks later identified persistent infection (PI) in 0.23% of the herd. Procedures followed the manufacturer’s protocol for result validation.

#### 2.1.2. PI Cow Screening

Three weeks later, serum and whole blood samples were collected again from the initially BVDV-positive cattle. Samples testing strongly positive in antigen ELISA were further analyzed using reverse transcription polymerase chain reaction (RT-PCR). Primers targeting the BVDV 5′UTR region (267 bp) were used:

Forward: 5′-CCTAGCCATGCCCTTAGTAGGACT-3′;

Reverse: 5′-GGAACTCCATGTGCCATGTACA-3′ [16].

### 2.2. Experimental Groups and Sampling

#### 2.2.1. Grouping

Eight female breeding cows (aged 12–18 months) were selected from a farm in northern Xinjiang. None had been vaccinated against BVDV. Four cows that were confirmed as BVDV-positive via serological ELISA and RT-PCR were assigned to the BVD_Ps group, while four BVDV-negative cows (BVD_Ng) served as healthy controls. The BVD_Ng cows had no history of BVDV infection.

#### 2.2.2. Sampling

Approximately 5 g of rectal fecal samples were collected using sterile polyethylene gloves, flash-frozen in liquid nitrogen, and stored for microbial analysis. Blood samples were concurrently collected from the tail veins for serological testing.

### 2.3. Gut Microbiota Analysis

#### 2.3.1. DNA Extraction

DNA was extracted from 200 mg of thawed feces using the QIAamp DNA Stool Mini Kit (Qiagen, Hilden, Germany). Following the manufacturer’s protocol, DNA was eluted in 200 μL of sterile ddH_2_O and stored at −20 °C. DNA concentration and integrity were verified using a Nanodrop 2000 spectrophotometer (Thermo Scientific, Waltham, MA, USA) and 1.0% agarose gel electrophoresis, respectively.

#### 2.3.2. 16S rRNA Gene Amplicon Sequencing

The V3–V4 region of bacterial 16S rRNA genes was amplified with the primers 341F (5′-CCTAYGGGRBGCASCAG-3′) and 806R (5′-GGACTACHVGGGTWTCTAAT-3′). Triplicate PCR reactions were performed using Pyrobest DNA Polymerase (DR500A, Takara, Shiga, Japan). Amplicons were purified with the AxyPrep DNA Gel Extraction Kit (AP-GX-500, Axygen Biosciences, Union City, CA, USA), quantified using a BioTek FLX800 microplate reader (Invitrogen Picogreen assay), and sequenced on an Illumina platform (Shenzhen Wekemo Tech Group Co., Ltd., Shenzhen, China) following the TruSeq DNA library preparation protocol.

#### 2.3.3. Bioinformatics

Raw sequences were processed in QIIME2 (v2021.8) using the DADA2 plugin for quality filtering, denoising, merging, chimera removal, and the generation of Operational Taxonomic Units (OTUs) [17]. Taxonomic assignment was performed using the Greengenes database (v13.8) [18]. The alpha (Faith’s PD) and beta (Bray–Curtis) diversity were calculated. LEfSe (v1.1.0) identified differentially abundant taxa (Kruskal–Wallis’s test, LDA score > 2.0) [19]. PICRUSt2 predicted metabolic pathways using MetaCyc and KEGG databases. Visualizations were generated in R (v3.6.0) with the circlize package (v0.4.8).

### 2.4. Statistical Analysis

Data are expressed as the mean ± SEM. Group comparisons were performed using independent *t*-tests (GraphPad Prism 10), Mann–Whitney tests (non-normal data), and Welch’s *t*-test (STAMP v2.1.3). Alpha diversity differences were assessed via the Wilcoxon test, while beta diversity was analyzed using PERMANOVA. All analyses retained outliers (e.g., BVD_Ng_1) to preserve data integrity. Significance was defined as *p* < 0.05.

## 3. Results

### 3.1. Epidemiological Survey and PI Cow Identification

**Figure 1:**
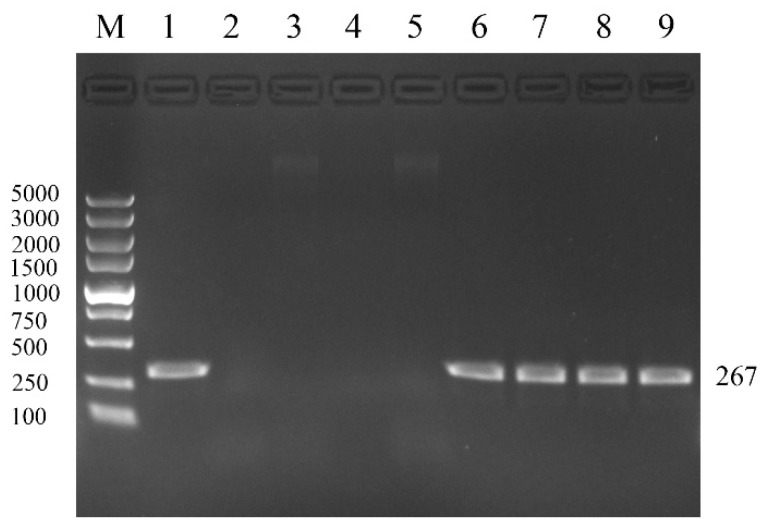
The identification of PI cows by RT-PCR.

The ELISA screening of 4310 cows revealed an initial BVDV antigen positivity rate of 0.44% (19/4310). Retesting 3 weeks later identified 10 persistently infected (PI) cows, yielding a herd-level PI prevalence of 0.23% (10/4310, Table 1). Four PI cows (BVD_Ps) and four BVDV-negative controls (BVD_Ng) were selected for further analysis, with RT-PCR confirming persistent infection in BVD_Ps (Figure 1).

M. DL5000 DNA Marker. 1. Positive control; 2–5. BVD-Ng samples; 6–9. BVD-Ps sample: The electrophoresis results of the amplified BVDV gene showed a length of 267 base pairs.

### 3.2. Gut Microbiota Composition

#### 3.2.1. OTU Distribution

**Figure 2:**
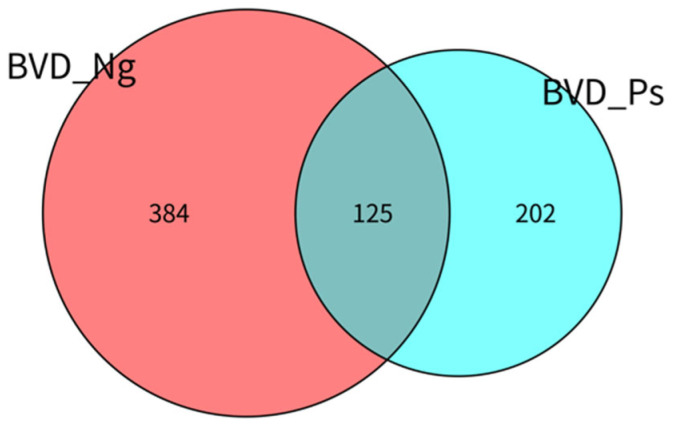
Venn diagram of OTUs of the gut microbiota between the BVD-Ng and BVD-Ps groups.

Venn analysis revealed distinct microbial communities between groups: BVD_Ng contained 384 unique OTUs (54% of total OTUs) and 125 shared OTUs (total: 509 OTUs), while BVD_Ps contained 202 unique OTUs (28.4% of total OTUs) and 125 shared OTUs (total: 327 OTUs). The 125 shared OTUs represented 17.6% of the combined total (711 OTUs) (Figure 2).

#### 3.2.2. Taxonomic Differences

**Figure 3:**
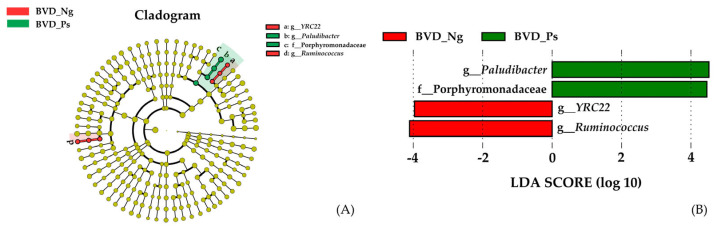
LEfSe analysis of differential gut microbiota between BVD-Ng and BVD-Ps groups (threshold = 3.0). (**A**): Cladogram and (**B**): LDA scores.

LEfSe analysis demonstrated *YRC22* and *Ruminococcus* as biomarkers in BVD_Ng, whereas *Paludibacter* (family *Porphyromonadaceae*) characterized BVD_Ps (LDA score of ≥3) (Figure 3A,B).

**Figure 4:**
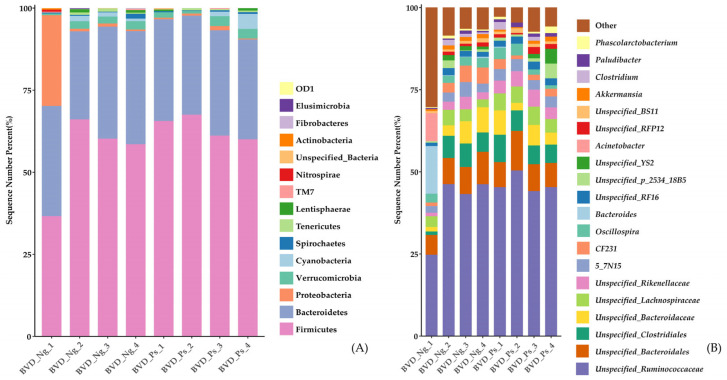
The relative abundance of gut microbiota in phyla (**A**) and genera ((**B**), top 20) between the BVD-Ng and BVD-Ps groups.

The relative abundances of microbial taxa in all BVD-Ng and BVD-Ps gut microbiota samples were analyzed at both the phylum and genus levels. The three dominant phyla—Firmicutes, Bacteroidetes, and Proteobacteria—collectively accounted for over 95% of the relative abundance in both groups (Figure 4A and Supplementary Table S1). Intergroup differences were assessed using the non-parametric Mann–Whitney U test, revealing no significant variations in phylum-level abundances between groups (all *p* > 0.05).

At the genus level, the top 20 most abundant taxa are shown in Figure 4B. Among 42 genera with a mean relative abundance ≥ 0.1% in either group (listed in Supplementary Table S2), *Ruminococcus* exhibited significantly higher abundance in the BVD-Ng group compared to BVD-Ps (*p* = 0.029).

### 3.3. Gut Microbiota Structural Differences

#### 3.3.1. Alpha Diversity

Analysis of alpha diversity indices (Chao1, Shannon, and Faith’s PD) revealed reduced microbial richness and evenness in the BVD-Ps group compared to BVD-Ng controls. Specifically, lower Chao1 and Shannon index values in BVD-Ps suggest decreased species richness and overall diversity, respectively. Notably, Faith’s PD—a phylogenetic diversity index—was significantly reduced in BVD-Ps (*p* < 0.05), indicating diminished evolutionary divergence among microbial taxa in infected cows (Table 2).

#### 3.3.2. Beta Diversity

**Figure 5:**
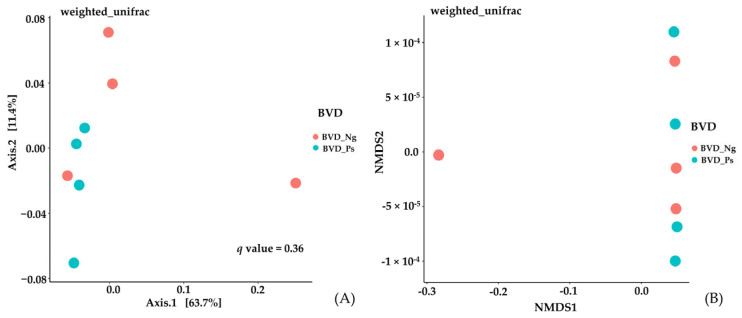
Beta diversity of gut microbiota by PCA plot (**A**) and NMDS ordination (**B**) between BVD-Ng and BVD-Ps groups.

Principal component analysis (PCA) of weighted UniFrac distances explained 63.7% (PC1) and 11.4% (PC2) of the variance, with no significant separation between groups (PERMANOVA: *p* > 0.05; Figure 5A). Similarly, NMDS ordination (stress = 0.12) demonstrated overlapping microbial community structures (Figure 5B), further supporting the absence of pronounced structural differences.

### 3.4. Metabolic Pathway Differences

**Figure 6:**
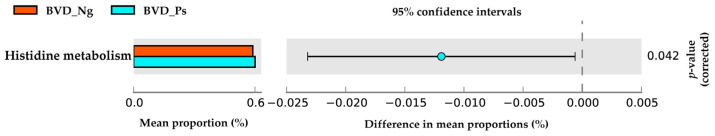
Differential abundance of the histidine metabolism pathway in KEGG between the BVD-Ng and BVD-Ps groups.

PICRUSt-predicted functional analysis identified histidine metabolism as the sole KEGG pathway differing significantly between groups, with elevated abundance in BVD-Ps (*p* < 0.05; Figure 6).

## 4. Discussion

### 4.1. Epidemiological Survey and PI Cow Screening

BVDV imposes substantial economic burdens on global cattle industries, primarily through persistent infection (PI) in fetuses exposed to non-cytopathic strains during gestation [20]. Transient (acute) and persistent BVDV infections cannot be differentiated by conventional virus isolation. Persistent infection is confirmed through antigen retesting after 3 weeks, a standard diagnostic approach for PI cattle. Our ELISA-based screening of 4310 cows in northern Xinjiang revealed a low initial BVDV positivity rate (0.44%), markedly lower than the 22.5–62.5% reported in 13 Xinjiang regions in 2020 [21]. This discrepancy likely reflects improved biosecurity protocols and immunization strategies—Farm A (3856 cows) implemented biannual BVDV inactivated vaccination, whereas Farm B (454 cows) lacked vaccination programs due to its remote location. Despite these efforts, sporadic infections persisted, underscoring the need for tailored immunization schedules that consider lactation status, gestation stage, and production purpose [22].

Three weeks later, the re-examination of 10 cows still showed positive results. Due to strict PI culling, enhanced monitoring, and environmental disinfection, the observed prevalence of PI (0.23%, Table 1) was lower than the 1–2% baseline reported by the World Organization for Animal Health [23].

### 4.2. Gut Microbiota Composition in PI Cows

Consistent with established mammalian gut microbiota profiles [24], the three dominant phyla—Firmicutes, Bacteroidetes, and Proteobacteria—collectively accounted for over 95% of the relative abundance in both groups (Figure 4A and Supplementary Table S1). While no phylum-level differences emerged, genus-level analysis identified *Ruminococcus* as significantly depleted in BVD-Ps (*p* < 0.05; Supplementary Table S2). LEfSe revealed *Paludibacter* (family Porphyromonadaceae) as a BVD-Ps biomarker, contrasting with *Ruminococcus* and *YRC22* enrichment in BVD-Ng (Figure 4A,B).

Notably, *Ruminococcus* abundance correlates positively with host weight in rabbits [25], while *YRC22* associates with enhanced *Mycoplasma* pneumoniae vaccine responses [26] and intestinal immunity when enriched by dietary mannan oligosaccharides [27]. Strikingly, BVD-Ps lacked Actinobacteria—a phylum pivotal for gut homeostasis [28]—but harbored increased *Paludibacter*, a putative butyrate producer linked to both colonic health [29,30] and ulcerative colitis [31]. The BVD-Ps group showed a deficiency of *Ruminococcus* in the gut microbiota of cows, a genus that plays a crucial role in rumen digestion and fermentation processes [32]. This suggests that viral infections could impair bovine rumen function. For example, bovine leukemia virus (BLV) infection was reported to reduce the abundance of rumen fermentation-related microbiota such as *Lachnospiraceae* and *Ruminococcaceae* [10], which aligned with the decreased relative abundance of *Ruminococcus* observed in BVDV-infected cows in this study. It was hypothesized that persistent BVDV infection might negatively impact rumen function in cows.

### 4.3. Microbial Diversity Alterations

Reduced alpha diversity in BVD-Ps (Chao1, Shannon, and Faith’s PD indices; Table 2) aligns with observations in CDV-infected pandas [33] and AIV-challenged poultry [34]. While beta diversity remained unchanged (Figure 5)—consistent with BLV infection patterns [10]—the diminished phylogenetic breadth (Faith’s PD) suggests reduced phylogenetic diversity within the microbial communities of PI cows.

### 4.4. Metabolic Pathway Implications

PICRUSt2 analysis revealed upregulated histidine metabolism in the BVD-Ps group (*p* < 0.05; Figure 6). Histidine exhibits vasodilatory effects and is closely linked to various allergic and inflammatory responses [35]. In a study by Yang et al., *Bifidobacterium animalis* BD400 alleviated systemic inflammation in collagen-induced arthritis (CIA) rats by modulating the gut microbiota structure and downregulating histidine metabolism, which concurrently restored intestinal permeability [36]. These findings suggest that gut microbiota may regulate histidine metabolism to mitigate host inflammatory responses. The elevated histidine metabolism observed in BVD-Ps cattle may thus reflect BVDV-associated gut inflammation, though mechanistic validation is needed.

### 4.5. Therapeutic Potential of Microbiota Modulation

Given BVDV’s immunosuppressive effects [37] and the gut microbiota’s immunomodulatory role [38], viral infections are known to induce significant alterations in gut microbiota composition, which reciprocally modulate interferon responses and shape immune defenses against viral pathogens [39]. Fecal microbiota transplantation (FMT) has gained attention as a therapeutic strategy for infectious and autoimmune diseases [40]. For example, FMT has proven effective against *Clostridioides difficile* infections [41] and shows promise in chronic hepatitis B management [42], while analogous approaches enhance resistance to African swine fever in pigs [43] and recovery from H9N2 in poultry [44]. Our findings suggest that microbiota-targeted approaches, such as probiotic supplementation or FMT, could complement existing BVDV control measures.

## 5. Conclusions

Persistent BVDV infection significantly reduced alpha diversity (species richness and phylogenetic breadth) without disturbing the overall structural stability of the gut microbiota. Key alterations included elevated *Paludibacter* abundance, decreased *Ruminococcus* levels, and an upregulated histidine metabolism pathway, suggesting potential associations with BVDV pathogenesis. It is worth conducting further mechanistic investigations into virus–microbiota–host interactions.

## Figures and Tables

**Table 1:** Screening results for cows persistently infected (PI) with bovine viral diarrhea virus.

Tested Samples	Positive Samples	Positive Rate (%)	Retested Samples	Positive in Retest	Positive Rate (%)
4310	19	0.44	15	10	0.23

**Table 2:** Index of α-diversity of gut microbiota between BVD-Ng and BVD-Ps groups.

Index of α-Diversity	BVD-Ng	BVD-Ps	*p* Value
Chao1	158.750 ± 21.554	102.750 ± 5.072	0.114
Shannon	6.601 ± 0.268	5.945 ± 0.112	0.200
Faith’s _pd	13.471 ± 0.722	10.240 ± 0.193	0.029

## Data Availability

Data are contained within this article and further inquiries can be directed to the corresponding authors.

## Supplementary Materials

The following supporting information can be downloaded at: https://www.mdpi.com/article/10.3390/vetsci12060538/s1. Table S1: The comparison of the relative abundance of Phyla in the gut microbiota between BVD-Ng and BVD-Ps group; Table S2: The comparison of the relative abundance of Genera in the gut microbiota between BVD-Ng and BVD-Ps group.
