# Supplementary material for: Fecal Microbiota Changes in Angus Beef Cows Persistently Infected by Bovine Viral Diarrhea Virus
Source: Vet Sci. 2025 Jun 2;12(6):538. doi: 10.3390/vetsci12060538 (PMC12197573; doi:10.3390/vetsci12060538)
Supplement: Supplementary file 1 [file vetsci-12-00538-s001.zip › Supplementary Table S1.pdf]

Supplementary Table S1 The comparison of the relative abundance of Phyla in the gut microbiota between BVD-Ng and BVD-Ps group

Phylum	The relative abundance (%)		
	BVD-Ng	BVD-Ps	*P* value
Firmicutes	55.349±6.457	63.574±1.784	0.665
Bacteroidetes	32.260±1.818	30.921±0.418	0.307
Proteobacteria	7.489±6.712	0.668±0.222	0.343
Cyanobacteria	0.924±0.350	1.482±1.073	0.928
Verrucomicrobia	1.840±0.452	2.077±0.547	0.686
Spirochaetes	0.556±0.304	0.388±0.066	0.886
Lentisphaerae	0.471±0.277	0.314±0.190	0.657
Tenericutes	0.494±0.223	0.394±0.149	0.971
Unspecified_Bacteria	0.040±0.026	0.098±0.098	0.999
TM7	0.154±0.089	0.074±0.074	0.429
Actinobacteria	0.110±0.221	0.000±0.000	0.356
Fibrobacteres	0.097±0.063	0.011±0.011	0.429

* Note: Only bacterial phyla with a mean relative abundance ≥0.1% in at least one group are listed in the table.

Non-parametric Mann-Whitney U test was used for analyzing intergroup differences.
