# Supplementary material for: Fecal Microbiota Changes in Angus Beef Cows Persistently Infected by Bovine Viral Diarrhea Virus
Source: Vet Sci. 2025 Jun 2;12(6):538. doi: 10.3390/vetsci12060538 (PMC12197573; doi:10.3390/vetsci12060538)
Supplement: Supplementary file 1 [file vetsci-12-00538-s001.zip › Supplementary Table S2.pdf]

Supplementary Table S2 The comparison of the relative abundance of Genera in the gut microbiota between BVD-Ng and BVD-Ps group

Genera	The relative abundance/%		
	BVD-Ng	BVD-Ps	*P* value
Unspecified_Ruminococcaceae	40.123±5.167	46.306±1.395	0.686
Unspecified_Bacteroidales	8.029±0.796	8.811±1.107	0.999
Unspecified_Clostridiales	5.196±1.408	6.442±0.636	0.999
Unspecified_Bacteroidaceae	4.749±1.491	4.916±1.186	0.999
Unspecified_Lachnospiraceae	3.493±0.487	4.955±0.313	0.057
Unspecified_Rikenellaceae	2.401±0.564	4.334±0.363	0.057
5_7N15	2.967±0.529	3.362±0.165	0.343
CF231	3.497±0.929	2.035±0.420	0.343
Oscillospira	2.574±0.138	2.434±0.635	0.999
Bacteroides	3.815±3.570	0.075±0.075	0.086
Unspecified_RF16	1.598±0.239	2.098±0.105	0.114
Unspecified_p_2534_18B5	0.952±0.492	1.769±0.909	0.343
Unspecified_YS2	0.924±0.350	1.482±1.073	0.999
Unspecified_RFP12	0.756±0.268	1.246±0.350	0.343
Unspecified_BS11	0.873±0.151	1.013±0.164	0.486
Akkermansia	1.055±0.182	0.820±0.242	0.686
Clostridium	0.798±0.366	0.871±0.552	0.999
Paludibacter	0.557±0.136	1.071±0.101	0.057
Phascolarctobacterium	0.538±0.122	0.809±0.425	0.886
Unspecified_S24_7	0.608±0.130	0.663±0.225	0.999
Unspecified_Christensenellaceae	0.506±0.287	0.731±0.465	0.999
Prevotella	0.503±0.106	0.446±0.215	0.686
Treponema	0.543±0.308	0.388±0.066	0.886
Unspecified_Victivallaceae	0.471±0.277	0.314±0.190	0.657
Unspecified_Desulfovibrionaceae	0.268±0.058	0.367±0.051	0.486
Unspecified_ML615J_28	0.183±0.106	0.281±0.105	0.657
Prevotella	0.385±0.235	0.058±0.038	0.486
BF311	0.253±0.132	0.182±0.027	0.886
Clostridium	0.248±0.248	0.158±0.158	0.999
Unspecified_Peptostreptococcaceae	0.210±0.210	0.180±0.180	0.999
Roseburia	0.382±0.221	0.000±0.000	0.429
Parabacteroides	0.350±0.327	0.031±0.031	0.714
Ruminococcus	0.324±0.065a	0.000±0.000b	0.029
Unspecified_RF39	0.205±0.124	0.113±0.113	0.714
rc4_4	0.106±0.013	0.166±0.057	0.343
Unspecified_Mogibacteriaceae	0.111±0.111	0.112±0.112	0.999
Unspecified_Paraprevotellaceae	0.175±0.045	0.044±0.044	0.086
Unspecified_Erysipelotrichaceae	0.124±0.072	0.069±0.069	0.999
Unspecified_F16	0.077±0.077	0.074±0.074	0.999
Unspecified_Bacteria	0.040±0.026	0.098±0.098	0.999
Sutterella	0.105±0.044	0.026±0.015	0.171
Fibrobacter	0.097±0.063	0.011±0.011	0.429

Note: Only bacterial genera with a mean relative abundance ≥0.1% in at least one group are listed in the table.

Non-parametric Mann-Whitney U test was used for analysis of intergroup differences.
